# Post-Thyroidectomy Hypocalcemia: A Single-Center Experience

**DOI:** 10.7759/cureus.20006

**Published:** 2021-11-29

**Authors:** Saad M Alqahtani, Amani S Alatawi, Yousef S Alalawi

**Affiliations:** 1 Department of Surgery, College of Medicine, Majmaah University, Al Majmaah, SAU; 2 Department of Surgery, King Fahad Specialist Hospital, Tabuk, SAU; 3 Department of Surgery, King Salman Armed Forces Hospital in North-Western Region, Tabuk, SAU

**Keywords:** thyroidectomy, temporary hypocalcemia, risk factors, post-thyroidectomy complications, persistent hypocalcemia

## Abstract

Introduction

Thyroidectomy is a frequent operation performed worldwide. The most common complication following thyroid surgery is hypocalcemia, caused by transient or persistent hypoparathyroidism. This study aimed to investigate the prevalence of hypocalcemia after thyroidectomy and to identify potential risk factors.

Methods

All thyroidectomies performed at a single tertiary center between 2012 and 2017 were retrospectively analyzed. Post-thyroidectomy hypocalcemia was evaluated in relation to risk factors such as age, sex, procedure type, and type of thyroid disease. Data were extracted from patient medical records. Patients with pre-operative hypocalcemia were excluded.

Results

We enrolled 182 patients who underwent thyroidectomy. Female patients comprised 83% (n = 151) of the total patients. Of all patients, 116 (63.7%) had developed post-operative temporary hypocalcemia and three (1.6%) had persistent hypocalcemia. Remarkably, no cases of mortality were reported. There was no significant relationship between the occurrence of hypocalcemia and age, type of thyroid disease, and sex. Conversely, there was a significant relationship between the development of hypocalcemia and the type of procedure (P < 0.001).

Conclusion

Thyroidectomy is a safe surgery with few complications when performed by a skilled surgeon. These complications result in longer hospital stays and higher costs. The most common post-thyroidectomy complication was hypocalcemia. Furthermore, patients who underwent total thyroidectomy were at the greatest risk of developing post-thyroidectomy hypocalcemia.

## Introduction

Thyroid disorders are among the most commonly occurring endocrine gland diseases worldwide. They can be treated either medically or surgically. Thyroidectomy (partial or total) is one of the most frequent operations performed globally [1,2]. Compression symptoms, suspected or known malignancy, presence of a solitary cold nodule in patients aged <20 years, cosmetic reasons, and the presence of a complex cyst or a cyst >4 cm in diameter are all indications for thyroidectomy [3].

Due to advancements in anesthesia, operative techniques and antisepsis, better surgical instruments, and understanding of thyroid anatomy and physiology, thyroid surgery is now considered a safe procedure [4,5]. However, complications following thyroid surgery may occur. These complications include hypocalcemia, recurrent laryngeal nerve injury, hematoma, seroma, stridor, loss of high-pitched voice, thoracic duct injury, wound infection, and tracheal injury [6]. Such complications occur less frequently when the surgery is performed by experienced surgeons (surgical volume of procedures performed per year) [3,7]. Hypocalcemia and recurrent laryngeal nerve injury are the most frequently encountered complications [8].

Post-thyroidectomy complications may be associated with some risk factors such as age, sex, increased gland size, type of thyroid disease, presence of fibrosis and inflammation, extent of thyroidectomy, and lymph node dissection [4]. According to a study conducted by Papaleontiou et al., advanced age, presence of comorbidities, and advanced disease are significant risk factors for post-thyroidectomy complications, especially in cases of thyroid cancer [9].

Our research group has previously published a paper on post-thyroidectomy complications in general [6]. The current study, however, focuses on the prevalence and risk factors associated with post-thyroidectomy hypocalcemia. Furthermore, we hope to share our experiences and compare our findings with those in the literature.

## Materials and methods

Methods

This retrospective study aimed to assess the prevalence of post-thyroidectomy hypocalcemia and to identify potential risk factors. It included all thyroidectomies performed between 2012 and 2017 at King Salman Armed Forces Hospital in North-Western Region, Tabuk, Saudi Arabia. Data were extracted from patient medical records. Among the entire group, 140 surgeries were performed by endocrine surgeons and 42 were performed by general surgeons. Post-thyroidectomy hypocalcemia was evaluated in relation to risk factors such as age, sex, procedure type, and type of thyroid disease. Clinical and biochemical evaluations were performed to diagnose hypocalcemia.

Statistical analysis

SPSS version 22 (Armonk, NY: IBM Corp.) was used for the data analysis. Categorical data are presented as numbers and percentages. They were analyzed using Pearson’s chi-square test and Fisher’s exact test. The Shapiro-Wilk test was performed to determine the normality of continuous data. Normally, distributed data are reported as mean ± standard deviation and a one-way analysis of variance was performed to analyze these data. The risk of having post-thyroidectomy hypocalcemia was examined using multivariate binary logistic regression analysis. This was based on the presence or absence of hypocalcemia and whether a total thyroidectomy was performed or not. Statistical significance was defined as a p-value of <0.05.

## Results

Among the 182 patients who underwent thyroidectomies, 105 (57.7%) had benign lesions and 77 (42.3%) had malignant lesions. The ages ranged between 15 and 95 years (mean 39.87 ± 12.67 years), with most patients being female (n = 151, 83%). Total thyroidectomy was the most common surgery performed (n = 107, 58.8%), followed by right hemithyroidectomy (n = 39, 21.4%) and left hemithyroidectomy (n = 24, 13.2%). Further, completion thyroidectomy and subtotal thyroidectomy were performed for 2.7% and 3.8% of the patients, respectively.

A total of 116 patients (63.7%) had temporary hypocalcemia and three developed persistent hypocalcemia (1.6%). The remaining patients (n = 63, 34.6%) did not develop hypocalcemia. Table 1 depicts the association between post-thyroidectomy hypocalcemia and various risk factors. In addition, there was no significant relationship between the occurrence of hypocalcemia and age, type of thyroid disease, and sex. Conversely, there was a significant relationship between the development of hypocalcemia and the type of procedure (P < 0.001). Temporary hypocalcemia was more common in patients who underwent total thyroidectomy (70.7%), followed by right hemithyroidectomy (15.5%) and left hemithyroidectomy (10.3%). Only three patients had permanent hypocalcemia after undergoing left hemithyroidectomy, subtotal thyroidectomy, or total thyroidectomy.

**Table 1:** Risk of hypocalcemia and associated risk factors The table is reproduced with permission from Alqahtani et al. [6]. *Statistically significant at P < 0.05.

			Post-thyroidectomy hypocalcemia			
			No (n = 63)	Temporary (n = 116)	Permanent (n = 3)	P-value
Age (years)	Mean		38.62	40.50	41.67	0.621
	Standard deviation		11.46	13.41	5.69	
Sex	Female	n	50	98	3	0.67
		%	79.4%	84.5%	100.0%	
	Male	n	13	18	0	
		%	20.6%	15.5%	0.0%	
Thyroid disease	Benign	n	38	65	2	0.84
		%	60.3%	56.0%	66.7%	
	Malignant	n	25	51	1	
		%	39.7%	44.0%	33.3%	
Type of procedure	Completion thyroidectomy	n	3	2	0	< 0.001*
		%	4.8%	1.7%	0.0%	
	Right hemithyroidectomy	n	21	18	0	
		%	33.3%	15.5%	0.0%	
	Left hemithyroidectomy	n	11	12	1	
		%	17.5%	10.3%	33.3%	
	Subtotal thyroidectomy	n	4	2	1	
		%	6.3%	1.7%	33.3%	
	Total thyroidectomy	n	24	82	1	
		%	38.1%	70.7%	33.3%	

A binary logistic regression model was utilized to evaluate the impact of demographic variables (namely age and sex), procedure type, and thyroid disease on the risk of developing post-thyroidectomy hypocalcemia. The model showed a statistically significant result (X^2^ = 20.69, P < 0.001). Furthermore, the model accurately identified 71.4% of patients with hypocalcemia and explained 15% of the hypocalcemia variation. Demographic variables and thyroid disease type had no meaningful contribution to the model (P > 0.05); however, the operation type, whether it was a total thyroidectomy or not, contributed significantly (P < 0.001). Among all the types of thyroidectomy, total thyroidectomy was the most common cause of hypocalcemia (odds ratio: 4.09) (Table 2).

**Table 2:** Binary logistic regression model The table is reproduced with permission from Alqahtani et al. [6]. *Statistically significant at P < 0.05

Regression model	Post-thyroidectomy hypocalcemia	
	P-value	Odds ratio
Age (years)	0.16	01.02
Sex	0.12	02.04
Thyroid disease	0.69	1.15
Total thyroidectomy	< 0.001*	04.09

## Discussion

Post-operative hypoparathyroidism is a common complication associated with prolonged hospitalization and higher costs [5]. It has been shown that the surgeon’s experience is associated with satisfying clinical and financial outcomes [7]. Transient or permanent hypoparathyroidism can lead to the development of post-operative hypocalcemia. According to the literature, the incidence of transient hypoparathyroidism and permanent hypoparathyroidism ranges from 0.3% to 49% and 0% to 13%, respectively [10]. Another study has reported that the risk of developing hypocalcemia can reach up to 63% [8]. Hypocalcemia after thyroidectomy is associated with the removal or devascularization of the parathyroid gland tissue [3]. In definitive hypocalcemia, the arterial or venous (or both) blood supply to the parathyroid glands is impaired. As a result, tetany can occur within 12 hours of surgery. Some authors argue that a single functioning parathyroid gland is sufficient to maintain normal gland activity. On the other hand, other authors believe that at least three parathyroid glands are required to restore normal function [8]. A devascularized parathyroid gland should be managed intraoperatively by reimplanting the fragmented pieces into the sternocleidomastoid muscle [8].

Temporary hypocalcemia is defined as a decrease in calcium levels following thyroidectomy that lasts for six to 12 months [11]. Our data showed that the incidence of post-thyroidectomy temporary hypocalcemia was 63.7%, which was treated with calcium and vitamin D supplementation. According to different studies, the incidence of temporary hypocalcemia is 43% and 63% [12,8]. Another study found that the incidence ranged between 50% and 68%, particularly after total thyroidectomy [11].

In contrast, permanent hypocalcemia is defined as a decrease in calcium levels after a total thyroidectomy lasting more than 12 months [11]. Our data showed that the risk of developing persistent hypocalcemia was 1.6%, which is consistent with the findings reported in previous studies [8,11]. Other studies have reported that up to 5% of patients had permanent hypocalcemia [12]. In our study, total thyroidectomy was the most common cause of hypocalcemia among all the types of thyroidectomy. A retrospective observational analysis showed that symptomatic hypocalcemia was more common following total thyroidectomy [8]. Moreover, subtotal thyroidectomy has been associated with a lower risk of hypoparathyroidism compared to total thyroidectomy [3,12].

Patients with low calcium levels before surgery are more likely to develop temporary post-thyroidectomy hypocalcemia. In addition, when calcium levels are ≤ 1.88 mmol/L within the first 24 hours after surgery, the risk of developing permanent hypocalcemia is increased [11]. Furthermore, a decrease in parathyroid hormone levels within the first 24 hours following surgery was a statistically significant predictor of developing post-thyroidectomy hypocalcemia (P < 0.001). Moreover, if the post-operative parathyroid hormone level dropped to 6-35 pg/mL within one hour to one day after surgery, the chance of developing temporary post-thyroidectomy hypocalcemia reached 69-100% [11].

Our study found that no significant relations between age, sex, and the risk of having hypocalcemia, as confirmed by a binary logistic regression model; however, older age and female sex were found to be significant risk factors for developing hypocalcemia following thyroidectomy in a multivariate analysis [11]. According to the literature, there is no evidence for the effect of age on the development of post-thyroidectomy hypocalcemia [11,13]. However, certain studies found that post-thyroidectomy temporary hypocalcemia is more frequent in younger patients [14], whereas other studies have reported it to be more common in the elderly [11].

Our findings revealed no correlation between thyroid disease (benign vs. malignant) and the risk of developing post-thyroidectomy hypocalcemia (P = 0.84). This contradicts the finding of a previous report by Pfleiderer et al. The authors found that the risk of hypocalcemia was higher in cases of toxic goiter and one-stage total thyroidectomy [12]. Furthermore, the incidence of definitive hypoparathyroidism was found to be slightly higher in cases of tumor pathology [8]. Several studies have investigated the factors associated with complications following thyroid surgery including age, increased gland size, sex, presence of fibrosis and inflammation, extent of thyroidectomy, and lymph node dissection [4]. In addition, extensive surgery, redo surgeries, and a surgeon’s experience all contribute to post-thyroidectomy hypocalcemia [3,15]. In Graves’ disease and redo surgeries, this could be attributed to the presence of adhesions between the thyroid gland capsule and the parathyroid gland [5].

Our study has several limitations. First, it was a single-center study with a small sample size. Second, it was retrospective in nature. Finally, data such as whether a central neck dissection was performed and whether redo surgeries were performed are lacking. To overcome these limitations, we intend to conduct a prospective multicenter study in the future.

## Conclusions

Thyroidectomy is a safe surgery with few complications when performed by a skilled surgeon. These complications result in longer hospital stays and higher costs. In this study, the risk of developing post-thyroidectomy hypocalcemia was higher in patients who underwent total thyroidectomy. However, there was no significant relationship between the occurrence of hypocalcemia and age, type of thyroid disease, and sex.
